# Preoperative chemoradiation with paclitaxel-carboplatin or with fluorouracil-oxaliplatin—folinic acid (FOLFOX) for resectable esophageal and junctional cancer: the PROTECT-1402, randomized phase 2 trial

**DOI:** 10.1186/s12885-016-2335-9

**Published:** 2016-05-18

**Authors:** Mathieu Messager, Xavier Mirabel, Emmanuelle Tresch, Amaury Paumier, Véronique Vendrely, Laetitia Dahan, Olivier Glehen, Frederique Vasseur, Thomas Lacornerie, Guillaume Piessen, Farid El Hajbi, William B. Robb, Stéphanie Clisant, Andrew Kramar, Christophe Mariette, Antoine Adenis

**Affiliations:** Department of Digestive and Oncological Surgery, University Hospital, Lille, France; University of Lille 2, Lille, France; FRench EsoGastric Tumours (FREGAT) Working Group, Lille, France; Department of Radiotherapy, Centre Oscar Lambret, Lille, France; Methodology and Biostatistics Unit, Centre Oscar Lambret, Lille, France; Department of Radiotherapy, Institut de Cancérologie de l’Ouest, Angers, France; Department of Radiotherapy, University Hospital, Bordeaux, France; Department of Gastroenterology, University Hospital, Marseille, France; Department of Digestive and Oncological Surgery, HCL Cancer Institute, Lyon, France; Unit of Medical Physics, Centre Oscar Lambret, Lille, France; SIte de Recherche Intégrée sur le Cancer (SIRIC) OncoLille, Lille, France; Department of Surgery, Beaumont Hospital, Dublin, Ireland; the Royal College of Surgeons in Ireland (RCSI), Dublin, Ireland; Clinical Research 18 Unit, Centre Oscar Lambret, Lille, France; Catholic University, Lille, France; Department of Gastrointestinal Oncology, Centre Oscar Lambret, Lille, France

**Keywords:** Esophageal cancer, FOLFOX, Paclitaxel-carboplatin, Chemoradiotherapy, Randomized trial

## Abstract

**Background:**

Often curative treatment for locally advanced resectable esophageal or gastro-esophageal junctional cancer consists of concurrent neoadjuvant radiotherapy and chemotherapy followed by surgery. Currently, one of the most commonly used chemotherapy regimens in this setting is a combination of a fluoropyrimidin and of a platinum analogue. Due to the promising results of the recent CROSS trial, another regimen combining paclitaxel and carboplatin is also widely used by European and American centers. No clinical study has shown the superiority of one treatment over the other. The objective of this Phase II study is to clarify clinical practice by comparing these two chemotherapy treatments. Our aim is to evaluate, in operable esophageal and gastro-esophageal junctional cancer, the complete resection rate and severe postoperative morbidity rate associated with these two neoadjuvant chemotherapeutic regimens (carboplatin-paclitaxel or fluorouracil-oxaliplatin-folinic acid) when each is combined with the radiation regime utilized in the CROSS trial.

**Methods/design:**

PROTECT is a prospective, randomized, multicenter, open arms, phase II trial. Eligible patients will have a histologically confirmed adenocarcinoma or squamous cell carcinoma and be treated with neoadjuvant radiochemotherapy followed by surgery for stage IIB or stage III resectable esophageal cancer. A total of 106 patients will be randomized to receive either 3 cycles of FOLFOX combined to concurrent radiotherapy (41.4 Grays) or carboplatin and paclitaxel with the same radiation regimen, using a 1:1 allocation ratio.

**Discussion:**

This ongoing trial offers the unique opportunity to compare two standards of chemotherapy delivered with a common regimen of preoperative radiation, in the setting of operable locally advanced esophageal or gastro-esophageal junctional tumors.

**Trial registration:**

NCT02359968 (ClinicalTrials.gov) (registration date: 9 FEB 2015), EudraCT: 2014-000649-62 (registration date: 10 FEB 2014)

## Background

According to the 2012 GLOBOCAN survey, esophageal cancer (EC) remains the 8^th^ most common cancer worldwide, with an estimated 456,000 new cases/year, and the 6^th^ most common cause of death from cancer with approximately 400,000 deaths/year [1]. Even after curative surgical treatment, the prognosis of EC is poor, with a 5-year survival rate of nearly 40 % in patients resected in a curative intent [2]. Because most patients will present with local and/or distant recurrences during follow-up, multimodal preoperative approaches, using neoadjuvant chemoradiation (nCRT) or neoadjuvant chemotherapy, have been introduced to improve outcomes. In locally advanced EC, meta-analysis has shown a significant survival benefit in favour of nCRT over surgery alone, as well as a trend favouring nCRT over neoadjuvant chemotherapy [3]. However, in clinical practice, results of this meta-analysis do not make the selection of one nCRT regimen over another any easier. Moreover, the radiation regimens used in the randomized trials pooled in the meta-analysis are heterogeneous, with inconsistencies in the total radiation dose used, the number of fractions delivered, the length of treatment, radiation field planning, dosimetry planning and quality control. Although most regimens were fluorouracil (FU) and cisplatin-based, heterogeneity in the chemotherapy combinations used as well as the number of cycles delivered also makes meaningful interpretation difficult. Consequently, many clinicians continue to use a chemotherapy regime of a fluoropyrimidin and a platinum analogue combined with concomitant radiotherapy.

The strongest evidence for the benefit of nCRT over surgery alone comes from the recently updated CROSS trial which compared nCRT with weekly carboplatin and paclitaxel for 5 weeks and concurrent radiotherapy (41.4Gy) to surgery only [4]. In this trial which included 366 patients with esophageal and gastro-esophageal junctional tumors the complete resection rate was increased from 69 % with surgery alone to 92 % with the combined therapy with no increase in the postoperative mortality rate [5]. Ultimately, the nCRT in this trial’s population provided a highly significant 34 % reduction of the risk of death, as well as a significant 42 % reduction of the risk of relapse [4]. Currently, a FU- and cisplatin-based nCRT regimen and the CROSS combination may both continue to be considered as a standard of treatment for locally advanced resectable esophageal and gastro-esophageal junctional cancers [6].

The aim of the PROTECT trial is to evaluate the complete resection rate and the severe postoperative morbidity rate of two different preoperative regimens. It will compare the use of a platinum analogue (oxaliplatin)- and FU-based regimen (FOLFOX), with a platinum analogue (carboplatin)- and paclitaxel-based regimen. Both chemotherapy regimens will be combined with concurrent radiation as per the CROSS protocol in patients with operable esophageal and gastro-esophageal junctional cancers.

## Methods/design

### Protocol overview

This randomized multicenter phase II trial will include patients with infra-carinal squamous cell carcinoma (SCC) or adenocarcinoma (ADC) of the esophagus and Siewert type I or II tumors of the gastro-esophageal junction who eligible for curative surgery. It compares two different preoperative chemotherapy regimens (FOLFOX or carboplatin-paclitaxel) both of which are combined with concomitant neoadjuvant radiotherapy in accordance with the CROSS regime.

### Inclusion criteria

Patients will be considered for inclusion if they conform to the following criteria:

- ✓ Resectable infra-carinal EC (beyond 25 cm from the incisors) or Siewert type I or II gastro-esophageal junctional cancers
- ✓ Invasive adeno or squamous cell carcinomas
- ✓ Stage IIB (T1 N1 M0 or T2 N1 M0) or stage III (T3 N1 M0 or T4 N0 N1 M0) tumors according to the 7^th^ Union for International Cancer Control (UICC) classification [7]
- ✓ Eastern Cooperative Oncology Group (ECOG) performance status (PS) 0, 1 or 2
- ✓ Patients must be eligible for preoperative chemoradiation with either FOLFOX or Paclitaxel-carboplatin
- ✓ Age ≥ 18 and ≤ 75 years
- ✓ Peripheral neuropathy ≤ grade 1 according to the NCI-CTC classification
- ✓ Adequate bone marrow, renal and liver function [neutrophil count ≥ 1500/mm^3^, platelet count ≥ 100 000/mm^3^, haemoglobin ≥ 10 g/dl (after transfusion, if required), creatinine < 15 mg/L, clearance of creatinine (Cockcroft formulae) ≥ 60 ml/min, prothrombin time ≥ 60 %, AST and ALT ≤ 2.5 × upper limit of normal, total bilirubin < 1.5 × upper limit of normal, normal serum albumin level)
- ✓ Start of treatment within 28 days after inclusion
- ✓ Negative pregnancy test (serum β-HCG) performed less than 1 week prior to the beginning treatment in females of reproductive age
- ✓ Patients must covered by government health insurance
- ✓ Patients must provide written informed consent

### Exclusion criteria

Any of the following will exclude patients from participation in the trial:

- Stage I, IIA or stage IV EC or Siewert 3 gastro-esophageal junctional tumors
- Contraindications for surgery related to patient comorbidities: PaO_2_ < 60 mmHg, PaCO_2_ > 45 mmHg, forced expiratory volume in one second < 1000 ml/s, cirrhosis, myocardial infarction or on-going coronary artery disease, severe peripheral arterial occlusive disease (≥ stage II of the Leriche-Fontaine classification), weight loss exceeding 15 % over a 6 months period, other serious illness or medical conditions (such as left ventricular failure or uncontrolled infection)
- Other malignant tumor within the last 5 years or synchronous malignant tumor
- Pre-menopausal patients not using adequate contraception
- Pregnant or breast-feeding woman
- Auditory disorders
- Other histological subtypes of EC or type I and II gastro-esophageal junctional tumors which are not either a SCC or ADC
- Tumors located at the pharyngo-esophageal junction, the cervical esophagus, supra-carinal esophageal tumors or type III gastro-esophageal junctional tumors
- Distant metastases, including metastasis to supra-clavicular nodes
- Recurrent laryngeal nerve palsy due to tumor invasion
- Tumor involvement of adjacent mediastinal structures
- Length and width of the tumor exceeding 8 and 5 cm, respectively
- Prior cervical, thoracic and/or abdominal radiotherapy with field overlapping the proposed radiotherapy field
- Tracheo-esophageal fistula or invasion of the tracheo-bronchial airway
- Any other synchronous experimental drug treatment
- Previous hyper sensibility reaction to compounds containing a fluoropyrimidin, platinum salt or a taxane
- Peripheral sensory neuropathy with functional impairment
- Yellow fever vaccination, prophylactic use of phenytoin, live-attenuated vaccines

### Endpoints

The primary endpoint is composite: it combines both the complete resection (R0) rate and the severe post-operative morbidity rate (grade ≥ 3) according to the Clavien-Dindo classification [8].

Secondary endpoints are i) tumor regression grade according to the Mandard classification [9] and complete pathological response (ypT0N0) rate, ii) survival outcomes with overall and disease-free survival measured from the date of randomization, iii) safety criteria of nCRT (number of adverse events, type of toxicities, and severity of adverse events graded using National Cancer Institute Common Terminology Criteria (NCI-CTC), version 4.0), (iv) 30-day postoperative mortality and morbidity according to the Clavien-Dindo classification, with focused interest on post-operative cardio-respiratory morbidities rates, v) quality of life assessed at baseline, the day before surgery, and every 6 months after surgery, until progressive disease or death, using the QLQC30 European Organisation for Research and Treatment of Cancer (EORTC) questionnaire, as well as the dedicated esophageal module OES18, vi) correlation between lung Dose-Volume-Histogram (DVH) and postoperative respiratory morbidity, and viii) tolerance of the preoperative regimen, estimated by the percentage of patients who will receive the whole therapeutic sequence without modification. Postoperative medical and surgical complications will be detailed using a standardized definition [10] and classified according to the Dindo-Clavien classification [8].

### Randomization

**Fig. 1:**
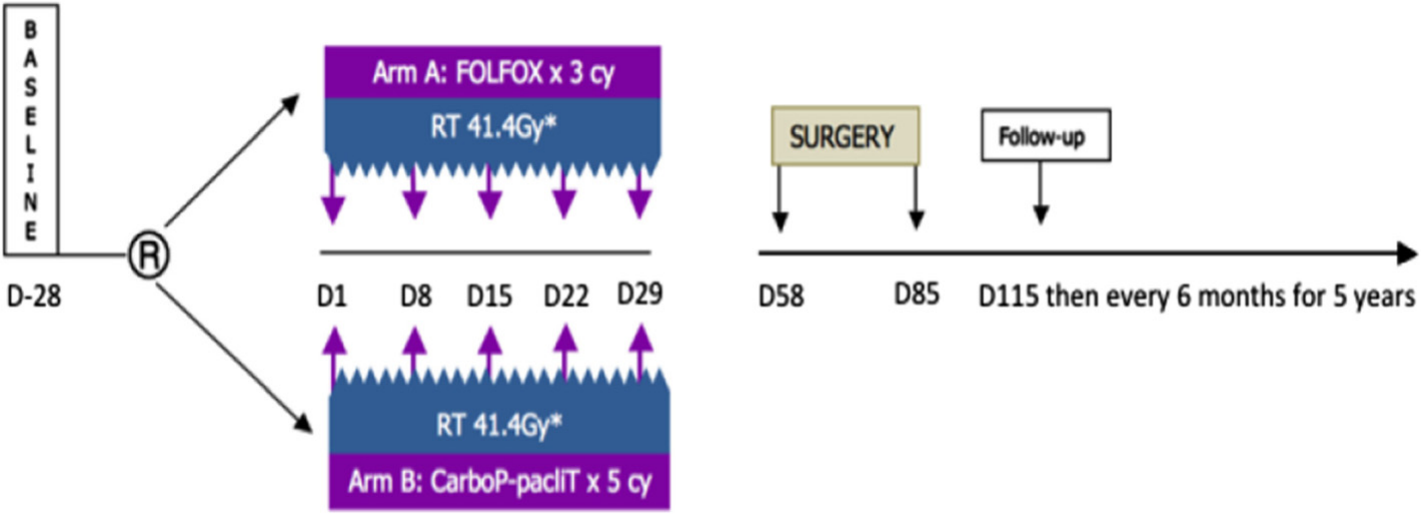
Study flow chart. R: randomization, cy: cycles, RT: radiotherapy, CarboP-pacliT: carboplatin-paclitaxel, FOLFOX: fluorouracil, leucovorin, oxaliplatin, *23 fractions of 1.8Gy, 5 fractions per week, starting the first day of the first cycle of chemotherapy

Patients will be allocated randomly either to arm A: FOLFOX + 41.4 Gy or arm B: Carboplatin-paclitaxel + 41.4 Gy (Fig. 1), in a 1:1 ratio. Randomization (CS-online tool, Clinsight) will be centrally performed with the minimization technique for the following stratification factors: ECOG PS (0 vs. 1–2), stage (IIB vs. III), histology (SCC vs. ADK), and centers.

### Pre therapeutic work-up

Baseline assessment (within 28 days prior to the start of treatment), will include: physical examination, measurement of weight, height, ECOG PS, standard laboratory tests (complete blood count, platelets, prothrombin rate, creatinine, AST-ALT, total bilirubin, albumin, serum β-HCG if applicable), ear, nose and throat examination, panendoscopy under general anaesthesia and a bronchoscopy with biopsies for patients with a SCC, esophagogastro-duodenoscopy with biopsies, laparoscopic exploration of the abdominal cavity to confirm the absence of contraindications for surgery (hepatic cirrhosis, hepatic or peritoneal metastases, or non-resectable tumor), a feeding jejunostomy may also be inserted during the laparoscopic procedure for preoperatively malnourished patients (more than 10 % of weight loss prior to trial inclusion), ultrasound exploration of the cervical area, a computed tomography (CT) scan of the neck, thorax, abdomen and pelvis, esophageal ultrasound endoscopy (EUS) examination, positon emission tomography (PET-CT) at the investigator’s discretion.

Clinical tumor staging (cTNM) will be based on data obtained from CT scan, EUS and PET-CT, and established using the 7^th^ UICC classification [7]. Quality of life questionnaires QLQC30 and OES18 module will be performed at baseline.

Before each cycle of chemotherapy, a physical examination, with documentation of adverse events will be performed, as well as standard laboratory tests. After the completion of nCRT tumor re-staging will be mandatory and will include at least an esophagoscopy with a minimum of four biopsies at the initial site of the tumor and a CT-scan of the neck, thorax, abdomen and pelvis.

### Treatment methods

Patients in arm A will receive 3 cycles of FOLFOX over 14 days. FU 400 mg/m^2^ will be given on Day 1 administered as a 10-min intravenous bolus dose followed by continuous intravenous infusion of 1600 mg/m^2^ over 2 days. Oxaliplatin 85 mg/m^2^, will be given as a 2-h intravenous infusion plus Folinic acid 200 mg/m^2^ (or calcium levofolinate 100 mg/m^2^) administered as a 2-h intravenous infusion.

Arm B will receive 5 cycles of carboplatin-paclitaxel. On days 1, 8, 15, 22 and 29, patients will receive Paclitaxel 50 mg/m^2^ and Carboplatin AUC 2 by intravenous infusion.

With regards to concomitant radiotherapy, patient position, definition of target volume and critical structures, simulation procedures and radiation technique will be similar in both arms and will match the description provided in the supplementary appendix of the CROSS trial [5]. A total dose of 41.4Gy will be given in 23 fractions of 1.8Gy, 5 fractions per week, starting the first day of the first cycle of chemotherapy. All patients will receive radiotherapy by external beam radiation using a 3-D-conformal radiation technique. Though the radiotherapy is done without intensity-modulated radiotherapy, treatment report will follow the International Commission on Radiation Units and Measurements 83 recommendations.

En bloc esophageal resection will be performed 4 to 8 weeks after the completion of nCRT through a transthoracic approach with a two field extended lymphadenectomy (Ivor Lewis procedure) and in accordance surgical quality criteria guidelines [11].

### Data collection and follow-up

The data management team of the Methodology and Biostatistics Unit of the Oscar Lambret Cancer Centre will undertake data Management. A trial-specific database will be created, tested and validated before the start of data capture. This database will be developed using Clinsight (ENNOV). The essential data necessary for monitoring the primary and secondary endpoints will be identified and managed at regular intervals throughout the trial in collaboration with the coordinator and the Oscar Lambret Cancer Centre Sponsorship Unit. The electronic case report forms will be completed at each investigator site. Treatment will continue unless one or more of the following occur i) disease progression during preoperative treatment (according to Response Evaluation Criteria in Solid Tumors, version 1.1 [12]), ii) death, iii) unacceptable toxicity, or, iv) withdrawal of consent for any other reason.

Outpatient examination will be performed 1 month after surgery (physical examination) and every 6 months thereafter (Quality of life: EORTC QLQC30 and OES18 modules) for 5 years. At each 6 month follow up all patients will have a clinical examination and thoraco-abdominal CT scan. Other investigations will be performed as required.

### Participating centers

Five centers (Lille, Bordeaux, Lyon, Nantes/Angers, Marseille; France) will participate in this study.

### Statistical analyses and sample size

This trial is based on a Bryant and Day two-stage design with stopping rules for unacceptable results for rates of R0 resections or excessive toxicity in terms of severe post-operative morbidity in each arm. According to the literature, the expected complete R0 resection rate should be at minimum 75 % and severe postoperative toxicity rate should be most 45 %. Fifty-three patients in each arm (48 evaluable) need to be included for a total of 106 patients according to following parameters:

- pR0 = 0.75 and pT0 = 0.55 corresponding respectively to an unacceptable R0 rate of ≤75 % and an unacceptable severe postoperative toxicity rate of 45 % or more.
- pR1 = 0.90 and pT1 = 0.75 corresponding to an acceptable R0 rate of 90 % or more, and an acceptable severe postoperative morbidity rate of 25 % or more, respectively.
- Errors rates αR = 0.10, αT = 0.10, and β = 0.15 corresponding respectively to a false positive error rate for response of 10 %, a false positive error rate for toxicity of 10 %, and 85 % power.
- Results will be evaluated after a first stage and the decision on continuing with one or both arms will be based on the following rules. In the first 20 patients in each arm, if there are ≤ 15 R0 resections or ≥ 9 patients with severe postoperative morbidity (≤11 patients without toxicity) then that treatment arm will not be considered to be of sufficient merit to consider further evaluation. If both treatment arms fail to satisfy the decision rules, then the trial will be stopped after this initial stage. If both treatment arms satisfy the decision rules, then the trial will continue to the second stage. If only one treatment arm satisfies the decision rules, then continuation of the trial with 1 or 2 arms will depend on the recommendations of the Data Monitoring Committee.
- During a second stage, each treatment will be evaluated after a total of 48 patients in each arm can be evaluated. Either arm will be considered of insufficient value if there are ≤ 39 R0 resections or ≥ 18 patients with severe post operative toxicity (≤30 patients without severe toxicity). Results at the end of the trial will allow a go/no go decision to be made for continuing to a phase III trial.

### Ethics and safety

The study protocol has been approved by our local ethics committee (CPP Nord-Ouest IV, November 4^th^, 2014), and by our national regulatory agency (Agence Nationale de Sécurité du Médicament et des Produits de Santé, August 29^th^, 2014).

## Discussion

The present trial offers the unique opportunity to compare 2 standards of chemotherapy delivered with a common regimen of preoperative radiation, in the setting of operable locally advanced esophageal or gastro-esophageal junctional tumors.

Paradoxically, before the CROSS trial publication in 2012 [5], evidence for a benefit of nCRT over surgery relied solely on randomized studies that mainly failed to demonstrate a survival benefit. Most of these trials were underpowered and the significant survival advantage of nCRT over surgery alone only appeared with meta-analysis [3]. As most of the nCRT regimens included in such meta-analysis were FU- and cisplatin-based, this regimen became a standard of care in many countries. Recent data, in the setting of definitive chemoradiation, suggested that the use oxaliplatin could be more convenient and safer, with less sudden death, compared to cisplatin [13]. Many oncologists have therefore changed their practice from using a nCRT regimen with FU and cisplatin, to a nCRT regimen with FOLFOX.

The carboplatin/paclitaxel combination is a key component of a nCRT regimen which has demonstrated a significant survival benefit over surgery alone, in the unique, well designed and adequately powered CROSS trial [4, 5]. However, this regimen has not been universally accepted into everyday practice, potentially because of selection bias which could have resulted in less nCRT-induced complications [6]. Indeed, most tumors in the CROSS trial arose from the lower third of the esophagus and gastro-esophageal junction and this usually correlates with less postoperative morbidity compared to upper third tumors. Moreover, the lung volume spared from radiation is greater in gastro-esophageal junctional tumors than in upper third cancers (a critical point in the development of radiation-induced pneumonitis and subsequent postoperative mortality). The favorable impact of the CROSS nCRT regimen may also be explained by a moderate total dose of radiation (41.4 Gy), smaller radial margins than in other trials, and modern dosimetry with 3D planning [5], which all improved the safety of treatment and of subsequent surgery. Further, the favorable impact of this regimen could possibly result from the fact that it did not include cisplatin, a compound that has been found to be associated with more sudden deaths [13], and more thromboembolic events [14] than oxaliplatin-based regimens.

Finally, as an exploratory study, we will analyse whether any relationship exists between the characteristics of the delivered radiotherapy, such as the dose delivered to lung tissue, and severe postoperative pulmonary toxicities. This question has been addressed in at least 2 retrospective studies [15, 16], without a consensus being reached on what characteristics of radiotherapy predict postoperative pulmonary toxicity. In the current ancillary study we will evaluate this putative relationship and in addition evaluate the role of both tumor location and the allocated preoperative chemotherapy regimen, as both of these parameters have been suspected to interact with postoperative pulmonary toxicity [17, 18]. The full DVH instead of thresholds will be analyzed to be able to find any correlation between dose, volume and toxicity.

## Conclusion

To summarize, in patients with resectable locally advanced esophageal and gastro-esophageal junctional cancers who are eligible for curative treatment, it is of great clinical interest to compare the two current standards of neoadjuvant chemotherapy (carboplatin/paclitaxel-based and oxaliplatin/FU-based) combined with a common standardized protocol for concomitant radiotherapy. Before performing a formal phase 3 trial, we first designed this randomized phase 2 trial to investigate the complete resection rate and the severe postoperative morbidity rate of these two regimens.

## Ethics approval and consent to participate

This study was performed according to the Declaration of Helsinki. Written consent was required from patients. The study protocol has been approved by our local ethics committee (Comité de Protection des Patients Nord-Ouest IV, reference number: 14/53, November 4^th^, 2014), and by our national regulatory agency (Agence Nationale de Sécurité du Médicament et des Produits de Santé, reference number: 140723A-12, August 29^th^, 2014).

## Consent for publication

Not applicable.

## Availability of data and materials

Non applicable (ongoing study).

## Abbreviations

ADC: adenocarcinoma
CT: computed tomography
DVH: Dose-Volume-Histogram
EC: esophageal cancer
ECOG: Eastern Cooperative Oncology Group
EORTC: European Organisation for Research and Treatment of Cancer
EUS: esophageal ultrasound endoscopy
FOLFOX: oxaliplatin and FU-based regimen
FU: fluorouracil
NCI-CTC: National Cancer Institute Common Terminology Criteria
nCRT: neoadjuvant chemoradiation
PET-CT: positon emission tomography
PS: performance status
R0: complete resection rate
SCC: squamous cell carcinoma
UICC: Union for International Cancer Control
